# Roles of exosomes and exosome-derived miRNAs in pulmonary fibrosis

**DOI:** 10.3389/fphar.2022.928933

**Published:** 2022-08-11

**Authors:** Yongfeng Yang, Hong Huang, Yi Li

**Affiliations:** ^1^ Precision Medicine Key Laboratory, Institute of Respiratory Health, West China Hospital, Sichuan University, Chengdu, Sichuan, China; ^2^ Key Laboratory of Transplantation Engineering and Immunology, Institute of Clinical Pathology, Ministry of Health, West China Hospital, Sichuan University, Chengdu, Sichuan, China

**Keywords:** exosome, pulmonary fibrosis, biomark, therapy tool, microRNA

## Abstract

Pulmonary fibrosis is a chronic, progressive fibrosing interstitial lung disease of unknown etiology that leads rapidly to death. It is characterized by the replacement of healthy tissue through an altered extracellular matrix and damage to the alveolar structure. New pharmacological treatments and biomarkers are needed for pulmonary fibrosis to ensure better outcomes and earlier diagnosis of patients. Exosomes are nanoscale vesicles released by nearly all cell types that play a central role as mediators of cell-to-cell communication. Moreover, exosomes are emerging as a crucial factor in antigen presentation, immune response, immunomodulation, inflammation, and cellular phenotypic transformation and have also shown promising therapeutic potential in pulmonary fibrosis. This review summarizes current knowledge of exosomes that may promote pulmonary fibrosis and be utilized for diagnostics and prognostics. In addition, the utilization of exosomes and their cargo miRNAs as novel therapeutics and their potential mechanisms are also discussed. This review aims to elucidate the role of exosomes in the pathogenesis of pulmonary fibrosis and paves the way for developing novel therapeutics for pulmonary fibrosis. Further in-depth research and clinical trials on this topic are encouraged in the future.

## Introduction

Exosomes are small extracellular vesicles generated by inward budding of the membrane into the lumen of the compartment (Koh et al., 2020). The diameter of exosomes ranges from 40 to 160 nm (Li et al., 2022), and these structures play an important role in intercellular communication by transferring nucleotides or proteins, which then act accordingly (Merckx et al., 2020). Various exosomes can be detected in various body fluids, including blood, urine (Makler and Asghar, 2020), synovial fluid, breast milk, ascites, thorax-related sputum, bronchoalveolar lavage fluid (BALF), and pleural effusions, which represent a unique tool to study the pathophysiology and biomarker discovery of respiratory diseases (Kadota et al., 2016; Lucchetti et al., 2021). Recently, exosomes have been recognized as a novel disease biomarker because they reflect the physiological state and microenvironment of the cell of origin, are readily found in body fluids, and are stable in the extracellular environment (Kok and Yu, 2020).

In respiratory medicine, there is increasing evidence regarding the involvement of exosomes in the pathogenesis of lung diseases, such as chronic obstructive pulmonary disease (COPD), asthma, alpha-1 antitrypsin deficiency (AATD), pulmonary fibrosis (PF), and lung cancer (Trappe et al., 2021). For example, exosomes are reported to be involved in inflammation and immune activation in asthmatic patients (Admyre et al., 2003). Furthermore, exosomes can also transfer microRNAs (miRNAs) that are capable of inducing disease phenotypes in COPD target cells (Fujita et al., 2015). Current research has predominantly focused on the role of exosomes in lung cancer. There are numerous published reports on the pathophysiological role of exosomes in cancer initiation, progression, invasion, metastasis, and new therapeutic approaches using exosomes as drug delivery systems (Xunian and Kalluri, 2020). In addition, the number and profiles of exosomes are altered according to the pathophysiological status of the disease; therefore, exosomes can be used as biomarkers to monitor disease (Zheng et al., 2018). On clinicaltrials.gov, studies using exosomes as diagnostic tests or a molecular cargo that delivers miRNAs and proteins are underway in several lung diseases, such as clinical studies of circulating tumor DNA and combined detection of exosomes to identify benign and malignant pulmonary nodules (NCT04182893) and vaccination assays with dendritic cell-derived exosomes loaded with tumor antigens in non-small-cell lung cancer (NSCLC) (NCT01159288). Murine *in vitro* and *in vivo* models have suggested the potential involvement of exosomes in PF (Kadota et al., 2021; Zhou et al., 2021), but a direct correlation has not been clarified.

This review discusses the role of exosomes in PF to elucidate their potential application as diagnostic and prognostic biomarkers and therapeutic targets.

## Exosomes

Exosomes are nanosized membrane-bound vesicles released from cells and transport lipids, proteins, and nucleic acids (including mRNA, miRNA, lncRNA, circular RNA, ribosomal RNA, tRNA, and DNA fragments) (Li et al., 2022). Exosomes were first discovered in circulation during sheep reticulocyte maturation (Pan et al., 1985), and subsequently in other biological fluids (Makler and Asghar, 2020) and cell culture supernatants. Exosomes and microvesicles are collectively referred to as extracellular vesicles. Exosomes derived from different cell sources share similar surface proteins, including tetraspanins CD9, CD63, CD81, and CD82, as well as Alix and TSG101 (Zhu et al., 2021), which are recognized and currently used as markers for exosomes. The formation and secretion of exosomes is regulated by Rab proteins (Ostrowski et al., 2010), the endosomal sorting complex required for transport proteins (Tamai et al., 2010), and intracellular Ca^2+^ levels (Kim et al., 2021). Exosomes also express cell surface proteins that are similar to their origin. For example, mesenchymal stem cell (MSC)-derived exosomes express CD29, CD44, CD73, CD90, and integrins so that they can adhere and fuse with circulating or distant resident cells (Szul et al., 2016).

Exosomes can bind to the surface of target cells (receptor cells) and enter directly or activate receptors on the target cell surface to perform biological functions such as mediating antigen presentation and immune regulation. Macrophage-derived exosomes contain major histocompatibility complex (MHC) class II and costimulatory molecules that play a role in antigen presentation and naive T cell priming (Ramachandra et al., 2010). Exosomes released from activated macrophages can enhance immune cell activity by delivering inflammatory cytokines such as tumor necrosis factor (TNF) (O'Neill and Quah, 2008), while exosomes released by T cells can target various different cells and induce immunomodulatory effects (Lindenbergh and Stoorvogel, 2018). In summary, exosomes are crucial for intercellular communication, immune responses, immunomodulation, inflammation, and the transformation of cellular phenotypes.

## Exosomes contribute to the pathogenesis of pulmonary fibrosis

PF is a chronic, progressive, and destructive lung disease characterized by the accumulation of fibroblasts/myofibroblasts, increased deposition of extracellular matrix, and decreased lung function (Richeldi et al., 2017). The etiology of PF is currently unknown. There is increasing evidence that exosomes contribute to the pathogenesis of pulmonary fibrosis. Makiguchi et al. (2016) found that miR-21-5p was elevated in serum exosomes during acute inflammation and chronic fibrosis in a bleomycin-induced PF mouse model. In addition, patients with PF and high levels of miR-21-5p had a significantly poorer prognosis over 30 months, indicating the potential of miR-21-5p as a prognostic biomarker for PF. Chen et al. (2022) demonstrated that exosomes derived from hypoxia-induced alveolar epithelial cells stimulated interstitial PF through a mechanism dependent on the lncRNA HOTAIRM1. Increased numbers of BALF exosomes were reported in mice with experimental PF as well as in patients with idiopathic PF (IPF). This was because exosomes carry fibrotic mediators, such as WNT5A, which lead to increased fibroblast proliferation (Martin-Medina et al., 2018). Lacedonia et al. (2021) reported that exosomal miRNAs let-7d and miR-16 were significantly downregulated in the serum of patients with IPF. Expression of let-7d was also repressed in exosomes derived from BALF of PF mice (Xie et al., 2020). Furthermore, numerous differentially expressed miRNAs were detected in the lung-tissue-derived exosomes of patients with IPF compared with non-smoking controls, and these data further revealed lung-specific miRNAs associated with chronic lung diseases that could serve as potential biomarkers or therapeutic targets (Kaur et al., 2021). Liu et al. (2018) found that miR-125b-5p, miR-128-3p, miR-21-5p, miR-100-5p, miR-140-3p, and miR-374b-5p were upregulated, while let-7d-5p, miR-103-3p, miR-27b-3p, and miR-30a-5p were downregulated in exosomes in BALF from patients with PF. In addition, in a miRNA branch of exosomes, miR-142-3p was significantly upregulated in both sputum and plasma from patients with PF (Guiot et al., 2019; Njock et al., 2019). In addition,miR-142-3p was also positively correlated with the percentage of sputum macrophages and negatively correlated with the percentage of sputum neutrophils in patients with PF (Guiot et al., 2020). Furthermore, miR-142-3p was inversely correlated with lung diffusing capacity for carbon monoxide/alveolar volume (Njock et al., 2019).

**FIGURE 1:**
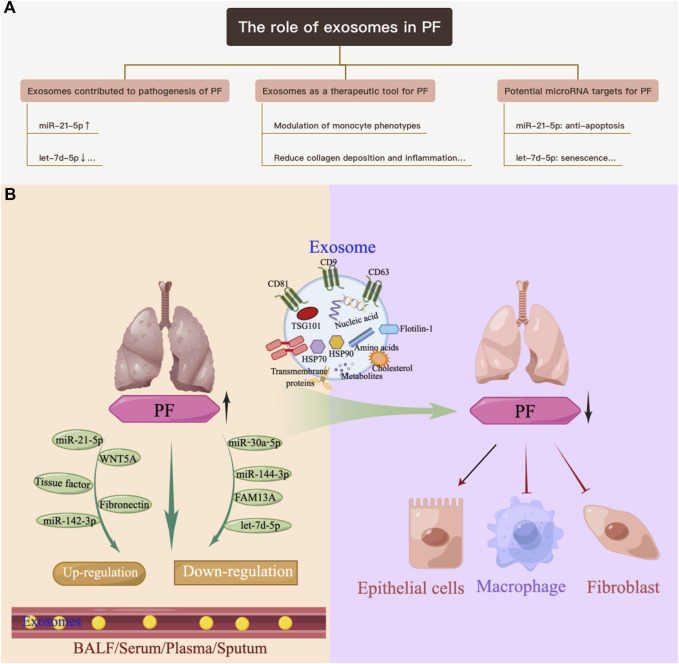
Overview of the role of exosomes in PF. **(A)** Exosomes contribute to the pathogenesis of PF. **(B)** Exosomes as a therapeutic tool for pulmonary PF.

Yao et al. (2019) demonstrated that M2 macrophage-derived exosomes overexpressed miR-328 and played a vital role in pulmonary fibroblast proliferation and the progression of PF by regulating FAM13A. Parimon et al. (2019) indicated that overexpressed Syndecan-1 in patients with PF, mainly in type II alveolar epithelial cells, was instrumental in controlling miRNA packaging in extracellular vesicles, including miR-144-3p, miR-142(a)-3p, miR-142b, miR-503-5p, and miR-34b-5p. Fibronectin expression on the surface of extracellular vesicles derived from fibroblasts of patients with PF could mediate their invasion, which may be related to the pathogenesis of fibrotic diseases (Chanda et al., 2019). Kang et al. (2019) revealed that extracellular vesicles derived from transforming growth factor-β (TGF-β)-stimulated fibroblasts contained PD-L1, which could inhibit T-cell proliferation and mediate fibroblast migration. Furthermore, fibroblast-derived extracellular vesicles contained increased levels of miR-23b-3p and miR-494-3p in PF, which induced epithelial cell phenotypic changes and were positively correlated with disease severity (Kadota et al., 2020). Kuse et al. (2020) revealed that miR-22 expression in exosomes from serum was increased and then decreased in a bleomycin-induced PF model. In addition, administration of the miR-22 mimic could ameliorate fibrosis by regulating fibroblast-to-myofibroblast differentiation. The identification of altered exosomes and elucidation of their role in the pathogenesis of PF can serve as references for the development of diagnostic biomarkers and subsequent therapeutic targets (Yamada, 2020; Hua et al., 2021; Yamada, 2021) (Table 1; Figure 1A).

**TABLE 1:** Exosomes associated with pulmonary fibrosis.

Author	Species	Exosome source	Outcome	Expression	Reference
Makiguchi	Human	Serum	miR-21-5p	↑	Makiguchi et al. (2016)
Chen	Mouse	Hypoxia-induced alveolar epithelial cells	HOTAIRM1	↑	Chen et al. (2022)
Martin-Medina	Human, Mouse	BALF	WNT5A	↑	Martin-Medina et al. (2018)
Lacedonia	Human	Serum	let-7d	↓	Lacedonia et al. (2021)
			miR-16	↓	
Xie	Mouse	BALF	let-7d	↓	Xie et al. (2020)
Liu	Human	BALF	miR-30a-5p	↓	Liu et al. (2018)
			let-7d-5p	↓	
			miR-103-3p	↓	
			miR-27b-3p	↓	
			miR-125b-5p	↑	
			miR-128-3p	↑	
			miR-21-5p	↑	
			miR-100-5p	↑	
			miR-140-3p	↑	
			miR-374b-5p	↑	
Guiot and Njock	Human	Sputum, plasma	miR-142-3p	↑	Guiot et al. (2019); Njock et al. (2019); Guiot et al. (2020)
		Plasma	miR-200c-5p	↑	Guiot et al. (2020)
		Sputum	miR-33a-5p	↑	Guiot et al. (2019); Njock et al. (2019); Guiot et al. (2020)
		Sputum	let-7d-5p	↓	Guiot et al. (2019); Njock et al. (2019); Guiot et al. (2020)
		Sputum	miR-192-5p	↑	Njock et al. (2019)
		Sputum	miR-26a-5p	↓	Njock et al. (2019)
		Sputum	miR-29b-3p	↓	Njock et al. (2019)
		Sputum	miR-423-3p	↓	Njock et al. (2019)
Yao	Rat	M2 macrophage	miR-328	↑	Yao et al. (2019)
			FAM13A	↓	
Parimon	Human Mouse	BALF	Syndecan-1	↑	Parimon et al. (2019)
			miR-144-3p	↓	
			miR-142(a)-3p	↓	
			miR-142b	↓	
			miR-503-3p	↓	
			miR-34b-5p	↓	
Chanda	Human	Fibroblast	Fibronectin	↑	Chanda et al. (2019)
Kang	Human	Fibroblast	PD-L1	↑	Kang et al. (2019)
Kadota	Human	Fibroblast	miR-23b-3p	↑	Kadota et al. (2020)
			miR-494-3p	↑	
			miR-19a-3p	↑	
			miR-127-3p	↑	
			miR-145-5p	↑	
			miR-424-5p	↑	
Kuse	Mouse	Serum	miR-22-3p	↑	Kuse et al. (2020)
			miR-16-5p	↑	
			miR-15a-5p	↑	
			miR-15b-5p	↑	
			miR-21a-5p	↑	
			miR-25-3p	↑	
			miR-93-5p	↑	
			miR-23a-3p	↑	
			miR-17-5p	↑	
			miR-29a-3p	↑	
			miR-32-3p	↓	
			miR-15a-3p	↓	
			miR-29c-5p	↓	
			miR-29b-1-5p	↓	
			miR-28a-3p	↓	
			miR-23b-5p	↓	
			miR-26a-1-3p	↓	
			miR-34a-3p	↓	
			miR-34c-5	↓	
			miR-21a-3p	↓	

## Exosomes as a therapeutic tool for pulmonary fibrosis

Recently, treatment aims for PF have been limited to prolonging life expectancy by slowing progression of the disease (Glass et al., 2022). Exosomes could improve management of PF and serve as an innovative therapeutic (Purghè et al., 2021). As early as 2014, Novelli and Neri’s team demonstrated that the BALF of patients with PF, which contained procoagulant microparticles and tissue factor, could activate coagulation factor X to Xa and potentially contribute to the pathogenesis of PF by regulating fibroblast growth and differentiation (Novelli et al., 2014). This team later reported that pirfenidone, one of only two U.S. Food and Drug Administration (FDA)-approved drugs for IPF at the time of writing, could inhibit p38-mediated generation of tissue factor in microparticles from H_2_O_2_ to stimulate alveolar epithelial cells (Neri et al., 2016). These findings showed that exosomes could be used to develop therapeutic applications.

Stem cells exhibit strong self-renewal and proliferation potential. Mesenchymal stem cells/mesenchymal stromal cells (MSCs) are multipotent stromal cells derived from the mesoderm and exhibit immunomodulatory, anti-inflammatory, and most importantly, antifibrotic properties. These properties are due, in part, to the activity of growth factors and cytokines secreted by the MSCs. Recently, researchers revealed that exosomes were responsible for the antifibrotic efficacy of MSCs (Fujita et al., 2018; Ma et al., 2022). MSCs derived from bone marrow (BM), adipose tissue (AD), and placenta confirmed that exosomes from MSCs could reduce inflammation by regulating related signaling pathways and polarization, and could also reduce collagen deposition in bleomycin- (Mansouri et al., 2019; Dinh et al., 2020; Wan et al., 2020), silica- (Choi et al., 2014; Phinney et al., 2015; Bandeira et al., 2018; Dinh et al., 2020), PM2.5- (Gao et al., 2020), and radiation- (Lei et al., 2020) induced PF models and TGF-β stimulated myofibroblasts (Shentu et al., 2017). The effect was also reported in stem cells from menstrual blood (Sun L. et al., 2019). However, Dinh et al. (2020) indicated that lung spheroid cell-derived exosomes exhibited superior therapeutic benefits in damage control and tissue repair compared with those from MSCs; moreover, the regenerative effects were more robust in the bleomycin model compared with the silica model. Guiot et al. (2020) showed that macrophage-derived exosomes alleviated fibrosis in airway epithelial cells and lung fibroblasts by delivering miR-142-3p, while Kadota et al. (2021) reported that human bronchial epithelial cell-derived extracellular vesicles (HBEC EVs) inhibited TGF-β-mediated induction of both myofibroblast differentiation and lung epithelial cellular senescence by attenuating WNT signaling. In the latter study, it was further suggested that administration of HBEC EVs was a promising antifibrotic modality of treatment for PF via miRNA-mediated inhibition of TGF-β-WNT crosstalk (Kadota et al., 2021). In addition, exosomes and their cargos, such as miRNAs, lncRNAs, and proteins, could promote or inhibit epithelial-mesenchymal transition (EMT), modulate the transformation of fibroblasts into myofibroblasts, contribute to the proliferation of fibroblasts, and promote immunoregulatory and mitochondrial damage during PF (Xie and Zeng, 2020). Thus, exosomes are emerging as a promising tool for the clinical benefit of cell therapy to treat PF and can potentially reduce the risks associated with cell transplantation (Table 2; Figure 1B).

**TABLE 2:** Application of exosomes in experimental models of pulmonary fibrosis.

Author	Cell source	Model	Target	Reference
Dinh	Lung spheroid cell, hBM-MSC	Bleomycin, silica	Regulate miR-99a-5p, miR-100-5p, miR-30a-3p, let-7 family	Dinh et al. (2020)
Mansouri	hBM-MSC	Bleomycin	Modulation of monocyte phenotypes	Mansouri et al. (2019)
Wan	hBM-MSC	Bleomycin	Regulate miR-29b-3p and frizzled 6; inhibit fibroblast proliferation, migration, invasion, and differentiation	Wan et al. (2020)
Choi	hBM-MSC	Silica	Reduce collagen deposition and inflammation	Choi et al. (2014)
Phinney	hBM-MSC, mBM-MSC	Silica	Regulate miR-451a, miR-1202, miR-630, miR-638; inhibit toll-like receptor signaling in macrophages	Phinney et al. (2015)
Shentu	hBM-MSC	TGF-β-stimulated myofibroblast	Regulate miR-199a/b-3p, miR-21-5p, miR-630, miR-22-3p, miR-196a-5p, miR-199b-5p, miR-34a-5p, and miR-148a-3p	Shentu et al. (2017)
Bandeira	hAD-MSC	Silica	Reduce collagen fiber content, size of granuloma, number of macrophages, and IL-1β, TGF-β	Bandeira et al. (2018)
Gao	hAD-MSC	PM2.5	Regulate let-7-5p, TGF-βR1; reduce apoptosis and necrosis, ROS, inflammation	Gao et al. (2020)
Lei	Placenta MSCs	Radiation	Regulate miR-214-3p, ATM/P53/P21; inhibit vascular damage, inflammation, and fibrosis	Lei et al. (2020)
Sun	Menstrual blood stem cells	Bleomycin	Regulate let-7-5p; regulates ROS, mtDNA damage, NLRP3 inflammasome activation	Sun L. et al. (2019)
Guiot	Macrophage	TGF-β stimulated cells	Regulate miR-142-3p, TGF-βR1	Guiot et al. (2020)
Kadoda	Human bronchial epithelial cell	Bleomycin	Inhibit TGF-β-mediated induction of both myofibroblast differentiation and lung epithelial cellular senescence by attenuating WNT signaling	Kadota et al. (2021)
Xie	Bone marrow mesenchymal stem cells (BMSCs)	Under pathological and physiological conditions	May promote or inhibit EMT of type II alveolar epithelial cells and the transformation of fibroblasts into myofibroblasts	Xie and Zeng, (2020)

## Potential miRNA targets for pulmonary fibrosis

### miR-21-5p: anti-apoptosis

Exosomal miRNAs were found to have potential applications in PF (Inomata et al., 2021; Peng et al., 2022; Yang et al., 2022). Since miR-21-5p is regarded as an oncogene in lung cancer, since the expression of miR-21-5p was significantly upregulated in patients with lung cancer, and it can be used as a biomarker for lung cancer (Zhou et al., 2022). Yan et al. (2018) reported that miR-21-5p inhibited TGF-βI to induce cell proliferation in NSCLC, while Tang et al. (2021) revealed that this miRNA boosted NSCLC progression by regulating SMAD7. Inhibition of miR-21-5p increased radiosensitivity in NSCLC (Song et al., 2017). The anti-apoptosis mechanism of miR-21-5p was subsequently investigated and was also found to play a role in many other lung diseases. Wu et al. (2022) indicated that ADMSC-EVs carrying miR-21-5p alleviated hyperoxia-induced lung injury (HILI) *via* the SKP2/Nr2f2/C/EBPα axis, and miR-21-5p could inhibit MAP2K3 expression and reduce cellular apoptosis in HILI (Qi et al., 2021). Liu et al. (2020) proved that miR-21-5p regulated hyperoxia-induced mitophagy and mitochondrial dysfunction by directly binding to the target gene PGAM5 (Liu et al., 2020). Moreover, miR-21-5p inhibited apoptosis of AEC II cells *via* PTEN/AKT in a hyperoxic acute lung injury rat model (Qin et al., 2019). Wang et al. (2018) demonstrated that resveratrol alleviated PF by regulating miR-21 through both the TGF-β1/SMAD and MAPK/AP-1 signaling pathways. Moreover, extracellular vesicles from MSCs pre-exposed to hypoxia exhibited increased miR-21-5p, which can promote lung cancer development by reducing apoptosis and promoting macrophage M2 polarization (Ren et al., 2019). In addition, exosomes from MSCs alleviated lung ischemia/reperfusion injury by delivering miR-21-5p targeting PTEN and PDCD4 (Ren et al., 2019). Administration of MSCs-derived exosomes or miR-21-5p agomir reduced pulmonary edema and dysfunction, M1 polarization of alveolar macrophages, and secretion of high mobility group box 1(HMGB1), IL-8, IL-1β, IL-6, IL-17, and TNF-α (Li et al., 2019). In conclusion, the above changes indicate a potential mechanism by which miR-21-5p regulates apoptotic/anti-apoptotic alterations in lung disease (Table 3).

**TABLE 3:** The roles of miR-21-5p in lung disease.

Author	Disease	Target/pathway	Role	Expression	Reference
Zhou	NSCLC	—	Biomarker	↑	Zhou et al. (2022)
Yan	NSCLC	TGF-βI	Induce cell proliferation	↑	Yan et al. (2018)
Tang	NSCLC	SMAD7	Boost NSCLC progression	↑	Tang et al. (2021)
Song	NSCLC	—	Decrease radiosensitivity	↑	Song et al. (2017)
Wu	HILI	SKP2/Nr2f2/C/EBPα axis	Alleviate HILI	—	Wu et al. (2022)
Qi	HILI	MAP2K3	Reduce cellular apoptosis	—	Qi et al. (2021)
Liu	HILI	PGAM5	Regulate hyperoxia-induced mitophagy and mitochondrial dysfunction	—	Liu et al. (2020)
Qin	Hyperoxic acute lung injury	PTEN/AKT	Inhibit apoptosis of AEC II cells	—	Qin et al. (2019)
Wang	PF	TGF-β1/SMAD and MAPK/AP-1 signaling pathways	Alleviate PF	—	Wang et al. (2018)
Ren	Lung cancer	PTEN, PDCD4, and RECK	Promote lung cancer development	—	Ren et al. (2019)
Li	Lung ischemia/reperfusion injury	PTEN and PDCD4	Alleviate lung ischemia/reperfusion injury	—	Li et al. (2019)

### Let-7d-5p: senescence

Previous studies revealed that let-7d-5p plays a key role in regulating the cell cycle and senescence, differentiation, and carcinogenesis (Markopoulos et al., 2017; Chen Y. N. et al., 2019). In addition, let-7d-5p affected the stemness and differentiation of MSCs, while transfection of fibroblasts with let-7d-5p reduced the expression of mesenchymal markers (Huleihel et al., 2014). In recent years, the role of let-7d-5p in degenerative diseases such as Alzheimer’s disease and amyotrophic lateral sclerosis has been investigated, and it was significantly downregulated in both diseases (Kumar et al., 2013; Mendes-Silva et al., 2016; Chen et al., 2018; Liguori et al., 2018). Moreover, let-7d-5p showed anti-inflammatory properties and inhibited intestinal epithelial cell apoptosis in necrotizing enterocolitis of neonatal rats by negatively regulating the LGALS3-dependent TLR4/NF-κB signaling pathway (Sun et al., 2020). The expression of let-7d-5p was downregulated both in the skin of systemic sclerosis and in the lungs of PF (Bagnato et al., 2017). Furthermore, when patients had both acute exacerbation and stable PF, let-7d-5p expression was downregulated compared with controls (Min et al., 2016). Significant downregulation of let-7d-5p was also observed in the serum of patients with NSCLC, and its expression could predict overall survival (Gasparini et al., 2015; Kumar et al., 2020). The fungus *Trametes robiniophila*, which is used as a traditional Chinese medicine, represses angiogenesis and tumor growth of lung cancer via strengthening let-7d-5p and targeting NAP1L1 (Gan et al., 2022). In contrast, overexpression of let-7d-5p was detected in cystic fibrosis (Ideozu et al., 2019), lung cysts/pneumothorax presentation of Birt-Hogg-Dubé Syndrome (Min et al., 2020), and chronic mucus hypersecretion in COPD (Tasena et al., 2018). Furthermore, ADSCs-EVs inhibited TGF-βRI by transferring let-7d-5p and further mitigated PF (Gao et al., 2020). Given that let-7d-5p is highly enriched in stem cells such as MSCs, therapies with cells or cell-free exosomes provide novel strategies for various diseases (Table 4).

**TABLE 4:** The roles of let-7d-5p in lung disease.

Author	Disease	Target/pathway	Role	Expression	Reference
Chen; Kumar; Mendes-Silva	Alzheimer’s diseaseAlzheimer’s disease	—	—	↓	Kumar et al. (2013); Mendes-Silva et al. (2016); Chen et al. (2018)
Liguori	Amyotrophic lateral sclerosis	—	—	↓	Liguori et al. (2018)
Sun	Necrotizing enterocolitis of neonatal rats	LGALS3-dependent TLR4/NF-κB signaling pathway	Anti-inflammatory properties and inhibited intestinal epithelial cell apoptosis	—	Sun et al. (2020)
Bagnato; Min	PF	—	—	↓	Min et al. (2016); Bagnato et al. (2017)
Gasparini; Kumar	NSCLC	—	Biomarker	↓	Gasparini et al. (2015); Kumar et al. (2020)
Gan	Lung cancer	NAP1L1	Repress angiogenesis	↓	Gan et al. (2022)
Ideozu	cystic fibrosis	—	—	↑	Ideozu et al. (2019)
Min	lung cysts/pneumothorax presentation of Birt-Hogg-Dubé Syndrome	—	—	↑	Min et al. (2020)
Tasena	COPD	—	—	↑	Tasena et al. (2018)
Gao	PF	TGF-ßRI	Mitigate PF	—	Gao et al. (2020)

### miR-100-5p: regulator of mammalian target of rapamycin

Accumulating studies have reported that miR-100 is a master regulator of PI3K/AKT/mTOR signaling in different diseases. The PI3K/AKT/mTOR signaling pathway is a critical regulator of cell growth and proliferation as well as stress responses. Wang et al. (2015) found that miR-100 regulated the proliferation of pulmonary artery smooth muscle cells in hypoxic pulmonary hypertension rats by inhibiting the expression of mTOR. Subsequently, miR-100-5p was confirmed to directly target the 3′-untranslated region (3′-UTR) of mTOR (Wu et al., 2019). Ye et al. (2015) reported that miR-100-5p promoted cell apoptosis and affected cell survival in amyloid *ß*-induced neuronal pathologies via the mTOR pathway, and Frith et al. (2018) demonstrated that miR-100-5p could modulate the fate of MSCs by altering mTOR signaling. A significant upregulation of mTOR was found in fibroproliferative diseases, suggesting mTOR inhibitors could be promising modulators of such diseases, including PF and liver fibrosis (Lawrence and Nho, 2018; Wang et al., 2019). Moreover, upregulation of miR-100-5p was also observed in PF and liver fibrosis (Peng et al., 2016; Liu et al., 2018), and it was assumed that miR-100-5p was a responsive factor rather than a pathogenic factor. Therefore, it is feasible that miR-100-5p could be utilized to repress mTOR expression and may be a potential therapy target for diseases. For example, miR-100-5p-abundant exosomes derived from MSCs provided a protective effect on articular cartilage and inhibited cell apoptosis in osteoarthritis (Wu et al., 2019). In a study by Dinh et al. (2020), miR-99a-5p and miR-100 were highly expressed in exosomes from lung spheroid cells and MSCs, and upregulated expression of miR-100 was identified in the exosomes of chronic PM2.5 exposure (Wang Y. C. et al., 2021). ReNcell-derived EVs inhibited hypoxia-induced proliferation, migration, and phenotype switching of pulmonary artery smooth muscle cells, at least in part, *via* the delivery of endogenous highly expressed miRNAs, let-7b-5p, miR-92b-3p, and miR-100-5p (Wang et al., 2020). These studies highlight that miR-100-5p can act as a potential target for fibroproliferative disease treatment (Table 5).

**TABLE 5:** The roles of miR-100-5p in lung disease.

Author	Disease	Target/pathway	Role	Expression	Reference
Wang	Hypoxic pulmonary hypertension	mTOR	Regulate the proliferation of pulmonary artery smooth muscle cells	—	Wang et al. (2015)
Ye	Amyloid *ß*-induced neuronal pathologies	mTOR pathway	Promote cell apoptosis and affect cell survival	—	Ye et al. (2015)
Frith	—	mTOR pathway	Modulate MSC fate		Frith et al. (2018)
Liu; Peng	PF and liver fibrosis	—	—	↑	Peng et al. (2016); Liu et al. (2018)
Wu	Osteoarthritis	mTOR	Provide a protective effect on articular cartilage and inhibit cell apoptosis	—	Wu et al. (2019)
Dinh	Exosomes from lung spheroid cells	—	—	↑	Dinh et al. (2020)
Wang	MSCs and chronic PM2.5 exposure	—	—	↑	Wang Y. et al. (2021)
Wang	—	—	Inhibit hypoxia-induced proliferation, migration, and phenotype switching of pulmonary artery smooth muscle cells	—	Wang et al. (2020)

### miR-142-3p: antifibrosis

It was reported that miR-142-3p is highly expressed in the lung interstitium during early lung development (Carraro et al., 2014). This miRNA positively regulates WNT/CTNNB1 (β-catenin) signaling by targeting adenomatous polyposis (APC), which is a negative regulator of WNT signaling (Isobe et al., 2014; Bartel et al., 2018). Impaired proliferation of parabronchial smooth muscle cell progenitors and premature differentiation occurred when miR-142-3p lost its function (Carraro et al., 2014). Aberrant expression of miR-142-3p in PF has been reported. However, the changes in miR-142-3p in patients with PF versus healthy controls are under debate (Guo et al., 2017; Njock et al., 2019; Parimon et al., 2019). WNT/β-catenin signaling has also been reported to be activated in PF. In experimental models, inhibition of WNT/β-catenin signaling reduces lung inflammation and fibrosis (Shi et al., 2017). All the research teams that conducted the above-mentioned studies believed that miR-142-3p was an antifibrotic miRNA. Moreover, Wang et al. (2016) found that overexpression of miR-142-3p suppressed the expression of profibrotic genes in cardiomyocytes by targeting HMGB1. In addition, Yang X. et al. (2017) revealed that plasma miR-142-3p levels were significantly decreased in patients with liver cirrhosis, and that miR-142-3p inhibited the TGF-β/SMAD signaling pathway to prevent hepatic stellate cell activation and reduce profibrotic markers. Zhu et al. (2018) demonstrated that overexpression of miR-142-3p attenuated high glucose-induced EMT in aortic endothelial cells by blocking the TGF-β1/SMAD signaling pathway in myocardial fibrosis, which confirmed the antifibrotic role of miR-142-3p. Consequently, Guiot et al. (2020) utilized miR-142-3p-enriched exosomes derived from macrophages to repress TGF-βR1, leading to antifibrotic properties in PF. However, contrary to these findings, some profibrotic conclusions of miR-142-3p have recently emerged, as reported by Cai et al. (2020). Thus, miR-142-3p warrants further investigation for future therapeutic use (Table 6).

**TABLE 6:** The roles of miR-142-3p in lung disease.

Author	Disease	Target/pathway	Role	Expression	Reference
Carraro	—	—	Early lung development	↑	Carraro et al. (2014)
Isobe; Bartel	Breast cancer; asthma	WNT/CTNNB1 (β-catenin)	Regulate the tumorigenicity	—	Isobe et al. (2014); Bartel et al. (2018)
Guo	PF	Cox-2	Inhibit apoptosis and inflammation	—	Guo et al. (2017)
Njock	IPF	—	—	↑	Njock et al. (2019)
Parimon	PF	—	—	↓	Parimon et al. (2019)
Wang	PF	High mobility group box 1	Suppress the expression of profibrotic genes	—	Wang et al. (2016)
Yang	Liver cirrhosis	TGF-β/SMAD signaling pathway	Prevent hepatic stellate cell activation and reduce profibrotic markers	—	Yang X. et al. (2017)
Zhu	Myocardial fibrosis	TGF-β1/SMAD signaling pathway	Attenuate high glucose-induced EMT in aortic endothelial cells	—	Zhu et al. (2018)
Guiot	PF	TGF-βR1	Antifibrotic properties	—	Guiot et al. (2020)
Cai	Myocardial fibrosis	—	Profibrotic properties	—	Cai et al. (2020)

### miR-29b-3p regulates collagen synthesis

The miR-29 family is considered a key regulator of tissue fibrosis, including heart, liver, lung, and kidney, as many reports have shown that members of this family inhibit collagen synthesis by directly binding to its 3ʹUTR in fibroblasts (He et al., 2013; Deng et al., 2017). Recently, accumulative reports demonstrated that miR-29b-3p regulated the TGF-β1/SMAD pathway in cardiac fibrosis (Liang et al., 2019; Xue et al., 2020). Moreover, the expression of miR-29b-3p was decreased in liver fibrosis, and miR-29b-3p overexpression repressed collagenous fibrosis and STAT3 (Tao et al., 2018; Gong et al., 2020). Lu et al. (2017) also reported that the lncRNA H19 promoted tendon differentiation by directly targeting miR-29b-3p, thus activating TGF-β1 and COL1A1 expression. In PF, downregulation of miR-29b-3p was detected (Mullenbrock et al., 2018), and downregulation of miR-29b promoted PF *via* the TGF-β1/SMAD pathway (Cushing et al., 2011; Pandit et al., 2011; Cushing et al., 2015). Conversely, overexpression of miR-29 prevented bleomycin-induced fibrosis as assessed by hydroxyproline content and collagen I mRNA expression (Cushing et al., 2015), suggesting that miR-29b might have a significant antifibrotic effect. In particular, miR-29b-3p-enriched BM-MSC exosomes suppressed fibroblast proliferation and decreased the level of hydroxyproline in the lung of a bleomycin-induced PF model (Wan et al., 2020). In summary, drugs targeting miR-29b-3p could be considered as potential therapeutics for fibroproliferative diseases (Table 7).

**TABLE 7:** The roles of miR-29b-3p in lung disease.

Author	Disease	Target/pathway	Role	Expression	Reference
Deng; He	Tissue fibrosis (including heart, liver, lung and kidney)	Binding to the 3ʹUTR	Inhibit collagen synthesist	—	He et al. (2013); Deng et al. (2017)
Liang; Xue	Cardiac fibrosis	TGF-β1/SMAD pathway	—	—	Liang et al. (2019); Xue et al. (2020)
Gong; Tao	Liver fibrosis	STAT3	Repress collagenous fibrosis	↓	Tao et al. (2018); Gong et al. (2020)
Lu	Liver fibrosis	TGF-β1	Activate COL1A1 expression	—	Lu et al. (2017)
Mullenbrock; Cushing; Pandit	PF	TGF-β1/SMAD pathway	Downregulate miR-29b and promote PF	↓	Cushing et al. (2011); Pandit et al. (2011); Cushing et al. (2015); Mullenbrock et al. (2018)
Wan	PF	—	Suppress fibroblast proliferation and decrease the level of hydroxyproline	—	Wan et al. (2020)

### miR-22-3p: tissue-specific regulator of fibrogenesis

MiR-22 has been proven to directly regulate bone morphogenic protein (BMP) by binding to its 3ʹUTR (Long et al., 2013). However, the effect of miR-22 on BMP seems to be tissue specific. For instance, an association between miR-22 and renal tubulointerstitial fibrosis was reported (Zhang et al., 2018), and similarly, miR-22 could promote the development of liver cirrhosis through BMP7 suppression in some studies (Ji et al., 2015; Zhou et al., 2018). The lncRNA Neat1 expedited the progression of liver fibrosis in mice through targeting miR-148a-3p and miR-22-3p to upregulate Cyth3 (Huang et al., 2021), and miR-30b-5p and miR-22-3p restrained fibrogenesis post-MI in mice *via* targeting PTAFR (Zhao et al., 2020). However, in contrast to these findings, downregulation of miR-22 increased the expression of collagen and fibrogenesis *in vitro*, whereas overexpression of miR-22 alleviated angiotensin II-induced cardiac fibrosis, indicating an antifibrotic effect of miR-22 (Hong et al., 2016). miR-22 expression was increased after bleomycin-induced PF in mice, while administration of an miR-22 mimic ameliorated lung lesions and decreased *α*-SMA expression (Kuse et al., 2020). Furthermore, miR-22 in MSC-derived extracellular vesicles was beneficial for PF (Shentu et al., 2017), demonstrating its antifibrotic effect in the lung. Due to the contrasting effects of miR-22 on fibrogenesis in different organs, local application of treatments is suggested to lessen the side effects of systemic administration (Table 8).

**TABLE 8:** The roles of miR-22-3p in lung disease.

Author	Disease	Target/pathway	Role	Expression	Reference
Long	Kidney fibrosis	Bone morphogenic protein (BMP)	Alleviate kidney fibrosis	—	Long et al. (2013)
Zhang	Renal tubulointerstitial fibrosis	—	—	—	Zhang et al. (2018)
Ji; Zhou	Liver fibrosis	BMP7	Promote the development of tissue cirrhosis	—	Ji et al. (2015); Zhou et al. (2018)
Huang	Liver fibrosis	Cyth3	Promote the development of tissue cirrhosis	—	Huang et al. (2021)
Zhao	Liver fibrosis	PTAFR	Promote the development of tissue cirrhosis	—	Zhao et al. (2020)
Hong	Cardiac fibrosis	—	Alleviate dangiotensin II-induced cardiac fibrosis	—	Hong et al. (2016)
Kuse; Shentu	PF	α-SMA	Antifibrotic effect in the lung	—	Shentu et al. (2017); Kuse et al. (2020)

### miR-15a: Hippo-YAP mediator

Growing evidence implicates miR-15a in the cell cycle and fibrotic diseases. Tijsen et al. (2014) found that inhibiting miR-15a exacerbated cardiac hypertrophy and fibrosis in mice by manipulating TGF-β. Inhibition of miR-15a/b also promoted fibrotic remodeling in type 2 diabetic hearts, whereas overexpression of miR-15a/b suppressed the activation of diabetic cardiac fibroblasts (Rawal et al., 2017). Furthermore, miR-15a/-15b, miR-18a-5p, miR-20a-5p, miR-26b-5p, miR-29, miR-133a, miR-141, miR-146, miR-200b, miR-203, miR-222, and miR-551b-5p were all downregulated in the diabetic heart and exhibited antifibrosis activity when they were overexpressed (Jin 2021). Fu et al. (2022) reported that miR-15a could inhibit LX-2 cell viability and hepatic fibrosis pathogenesis by targeting SOX9. However, the role and mechanism of miR-15a in PF remain controversial. MiR-15a was one of 161 miRNAs that were previously reported to be differentially expressed in the lungs of bleomycin-treated and control mice (Xie et al., 2011). Recently, miR-15a was discovered to be one of the most important miRNAs regulating the Hippo pathway, and knockdown of miR-15a promoted Twist expression by targeting YAP1, resulting in fibroblast activation and lung fibrosis (Chen Y. et al., 2019). In contrast, the lncRNA PFAR was proven to participate in PF by binding to and sponging miR-15a and by regulating the expression of YAP1 (Sun J. et al., 2019). Restoration of miR-15a can inhibit fibrogenesis (Chen Y. et al., 2019; Sun J. et al., 2019). However, according to Kuse et al. (2020), the expression of miR-15a in exosomes was strongly upregulated in bleomycin-induced PF. The reason for this discrepancy has yet to be elucidated; therefore, the use of miR-15a, especially that from exosomes, as a diagnostic and therapeutic target remains to be determined (Table 9).

**TABLE 9:** The roles of miR-15a in lung disease.

Author	Disease	Target/pathway	Role	Expression	Reference
Tijsen	Cardiac hypertrophy and fibrosis	TGF-β	Exacerbate cardiac hypertrophy and fibrosis	—	Tijsen et al. (2014)
Rawal; Jin	Diabetic cardiac fibroblasts	—	Suppress the activation of diabetic cardiac fibroblasts	↓	Rawal et al. (2017); Jin (2021)
Fu	Hepatic fibrosis	SOX9	Inhibit LX-2 cell viability and hepatic fibrosis	—	Fu et al. (2022)
Chen; Sun	PF	YAP1	Inhibit the activation of fibroblasts	↓	Chen Y. et al. (2019); Sun J. et al. (2019)
Kuse	PF	—	—	↑	Kuse et al. (2020)

### miR-23 regulates epithelial–mesenchymal transition

MiR-23 belongs to the miR-23/24/27 cluster and is highly correlated with cell proliferation, differentiation, invasion, migration, and EMT in cancer (Cao et al., 2012; Zheng et al., 2014). Previous research demonstrated that miR-23 was significantly increased in high glucose-treated EMT in mesothelial peritoneal cells, and inhibition of miR-23 attenuated the process of EMT (Yang L. et al., 2017). Similarly, knockdown of miR-23b could reverse TGF-β-induced liver fibrosis by regulating COLA1 and ACTA2 expression and stellate cell activation (Rogler et al., 2017). Aberrant expression of miR-23a-3p, miR-23b-3p, and miR-23b-5p was recently detected in PF (Kuse et al., 2020). However, exosomal miR-23a inhibited myofibroblast differentiation through inhibition of the TGF-β/SMAD pathway during wound healing (Fang et al., 2016), and was thus used in the experimental treatment of PF (Tan et al., 2018). Moreover, extracellular vesicles derived from umbilical cord MSCs enriched with miR-23 alleviated PF by inhibition of TGF-β signaling (Shi et al., 2021). The precise effect and mechanism of the miR-23 family requires further investigation (Table 10).

**TABLE 10:** The roles of let-7d-5p in lung disease.

Author	Disease	Target/pathway	Role	Expression	Reference
Cao; Zheng	Cancer	EMT	Correlate with cell proliferation, differentiation, invasion, migration	—	Cao et al. (2012); Zheng et al. (2014)
Yang; Rogler	Liver fibrosis	COLA1 and ACTA2	Reverse TGF-β-induced liver fibrosis	↓	Yang L. et al. (2017); Rogler et al. (2017)
Kuse; Fang; Tan; Shi	PF	TGF-β/SMAD pathway	Alleviate PF	—	Fang et al. (2016); Tan et al. (2018); Kuse et al. (2020); Shi et al. (2021)

## Discussion and future perspectives

Interest in the contribution of exosomes to the maintenance of lung homeostasis and the progression of PF, as well as the potential therapeutic utilization of exosomes, has increased substantially in recent years. Exosomes are critical for intercellular communication, immune response, immune regulation, inflammation, and cell phenotype transformation, and play a vital role in PF. This review summarized recent advances regarding the status of exosomes as potential biomarkers and therapeutic tools in PF. The potential involvement of exosomes in the pathological process of PF was first reviewed by describing the relevant characteristics of exosomes in PF, which highlights the potential value of exosomes as PF markers and therapeutic targets. Second, mouse *in vivo* and *in vitro* models were reviewed to demonstrate that multiple sources of exosomes have therapeutic effects on PF. These studies have collectively indicated that exosomes can be used as a therapeutic tool for PF and can reduce the risk associated with cell engraftment. Furthermore, exosomes have the potential to develop cell-free therapies. The main contents of exosomes are discussed, and the potential miRNA targets of PF are enumerated to highlight future research directions for elucidating the mechanism of exosomes in the treatment of PF. Exploration of the functional properties of exosomes in the context of PF could also reveal new avenues for therapeutic approaches.

Exosomes have potential clinical application value as a therapeutic tool for PF. However, several issues need to be addressed before they can be used in clinics. First, the characteristics and functions of exosomes derived from multiple types of cells should be extensively studied for safety aspects in future clinical applications. Exosomes can target several signaling pathways and molecules and therefore may induce previously unknown effects. Furthermore, treatments aimed at specific targets can cause persistent or lethal outcomes. Thus, thorough investigations of exosomes should be conducted to provide comprehensive information. Second, the exosomes need to be delivered with precision. Identification of target cells and limiting the destination to specific cells, for instance, fibroblasts/myofibroblasts, abnormal alveolar epithelial cells or immune cells in PF, should be performed to accurately interpret and correct the biological function. Finally, a manufacturing practice-grade standard protocol for the isolation and utilization of cell-free exosomes should be proposed. For the use of exosomes as diagnostic, prognostic, and therapeutic targets, the related protocols should be optimized and standardized to minimize variations due to technical issues.

There are some limitations to this review. First, when discussing exosomes as a potential therapeutic tool for PF, the mechanism of action was not explored in depth; only the potential miRNA targets were listed, and the description focused on the role of exosomes through miRNAs. Second, the review does not systematically discuss the major cells in PF that are altered by exosomes—fibroblasts, epithelial cells, and macrophages—so the specific cellular targets of exosomes in PF remain unclear, and further discussion on the mechanism of action is needed. In addition, each miRNA is known to have multiple targets. For example, miR-21-5p could alleviate HILI via the SKP2/Nr2f2/C/EBPα axis (Wu et al., 2022), PTEN/AKT signaling (Qin et al., 2019), inhibition of MAP2K3 (Qi et al., 2021) or by directly binding to the target gene PGAM5 (Liu et al., 2020). Although a single miRNA can often regulate multiple targets within a pathway or network, there are also risks of unpredictable and opposite effects on additional miRNA targets. Sun et al. (2015) demonstrated that miR-214 mediates cardiac fibroblast proliferation and collagen synthesis via inhibition of Mfn2 and activation of ERK1/2 MAPK signaling, but Dong et al. (2016) reported that miR-214 exerts cardio-protective effects by inhibition of fibrosis, and the inhibitory effect involves TGF-β1 suppression and MMP-1/TIMP-1 regulation. It is therefore feasible that the use of miRNA for disease therapy might have adverse implications for other essential biological pathways. Before clinical application in PF, further experiments are needed to explore the targets of miRNAs and their mimics *in vivo* and to determine their effects on other signaling pathways to avoid potential adverse effects.

## Abbreviations

AATD, alpha-1 antitrypsin deficiency; AD, adipose tissue; ADSCs, adipose mesenchymal stem cells; BALF, bronchoalveolar lavage fluid; BM, bone marrow; BMP, bone morphogenic protein; COPD, chronic obstructive pulmonary disease; ECM, extracellular matrix; EMT, epithelial–mesenchymal transition; EVs, extracellular vesicles; HBE, Chuman bronchial epithelial cell-derived; HILI, hyperoxia-induced lung injury; IPF, idiopathic pulmonary fibrosis; MHC, major histocompatibility complex; MSCs, mesenchymal stem cells/mesenchymal stromal cells; mTOR, mammalian target of rapamycin; NSCLC, non-small-cell lung cancer; PF, pulmonary fibrosis; TGF-β, transforming growth factor-β; TNF, tumor necrosis factor; 3′UTR, 3′untranslated region.

## References

[B1] Admyre C. Grunewald J. Thyberg J. Gripenbäck S. Tornling G. Eklund A. (2003). Exosomes with major histocompatibility complex class II and co-stimulatory molecules are present in human BAL fluid. Eur. Respir. J. 22 (4), 578–583. 10.1183/09031936.03.00041703 14582906

[B2] Bagnato G. Roberts W. N. Roman J. Gangemi S. (2017). A systematic review of overlapping microRNA patterns in systemic sclerosis and idiopathic pulmonary fibrosis. Eur. Respir. Rev. 26 (144), 160125. 10.1183/16000617.0125-2016 28515040 PMC9488120

[B3] Bandeira E. Oliveira H. Silva J. D. Menna-Barreto R. F. S. Takyia C. M. Suk J. S. (2018). Therapeutic effects of adipose-tissue-derived mesenchymal stromal cells and their extracellular vesicles in experimental silicosis. Respir. Res. 19 (1), 104. 10.1186/s12931-018-0802-3 29843724 PMC5975461

[B4] Bartel S. Carraro G. Alessandrini F. Krauss-Etschmann S. Ricciardolo F. L. M. Bellusci S. (2018). miR-142-3p is associated with aberrant WNT signaling during airway remodeling in asthma. Am. J. Physiol. Lung Cell. Mol. Physiol. 315 (2), L328–L333. 10.1152/ajplung.00113.2018 29722559

[B5] Cai L. Chao G. Li W. Zhu J. Li F. Qi B. (2020). Activated CD4(+) T cells-derived exosomal miR-142-3p boosts post-ischemic ventricular remodeling by activating myofibroblast. Aging (Albany NY) 12 (8), 7380–7396. 10.18632/aging.103084 32327611 PMC7202529

[B6] Cao M. Seike M. Soeno C. Mizutani H. Kitamura K. Minegishi Y. (2012). MiR-23a regulates TGF-β-induced epithelial-mesenchymal transition by targeting E-cadherin in lung cancer cells. Int. J. Oncol. 41 (3), 869–875. 10.3892/ijo.2012.1535 22752005 PMC3582905

[B7] Carraro G. Shrestha A. Rostkovius J. Contreras A. Chao C. M. El Agha E. (2014). miR-142-3p balances proliferation and differentiation of mesenchymal cells during lung development. Development 141 (6), 1272–1281. 10.1242/dev.105908 24553287 PMC3943182

[B8] Chanda D. Otoupalova E. Hough K. P. Locy M. L. Bernard K. Deshane J. S. (2019). Fibronectin on the surface of extracellular vesicles mediates fibroblast invasion. Am. J. Respir. Cell Mol. Biol. 60 (3), 279–288. 10.1165/rcmb.2018-0062OC 30321056 PMC6397976

[B9] Chen J. Qi Y. Liu C. F. Lu J. M. Shi J. Shi Y. (2018). MicroRNA expression data analysis to identify key miRNAs associated with Alzheimer’s disease. J. Gene Med. 20 (6), e3014. 10.1002/jgm.3014 29543360

[B10] Chen L. Yang Y. Yue R. Peng X. Yu H. Huang X. (2022). Exosomes derived from hypoxia-induced alveolar epithelial cells stimulate interstitial pulmonary fibrosis through a HOTAIRM1-dependent mechanism. Lab. Invest online ahead of print. 10.1038/s41374-022-00782-y 35477975

[B11] Chen Y. N. Ren C. C. Yang L. Nai M. M. Xu Y. M. Zhang F. (2019). MicroRNA let-7d-5p rescues ovarian cancer cell apoptosis and restores chemosensitivity by regulating the p53 signaling pathway via HMGA1. Int. J. Oncol. 54 (5), 1771–1784. 10.3892/ijo.2019.4731 30816441

[B12] Chen Y. Zhao X. Sun J. Su W. Zhang L. Li Y. (2019). YAP1/Twist promotes fibroblast activation and lung fibrosis that conferred by miR-15a loss in IPF. Cell Death Differ. 26 (9), 1832–1844. 10.1038/s41418-018-0250-0 30644438 PMC6748107

[B13] Choi M. Ban T. Rhim T. (2014). Therapeutic use of stem cell transplantation for cell replacement or cytoprotective effect of microvesicle released from mesenchymal stem cell. Mol. Cells 37 (2), 133–139. 10.14348/molcells.2014.2317 24598998 PMC3935626

[B14] Cushing L. Kuang P. Lü J. (2015). The role of miR-29 in pulmonary fibrosis. Biochem. Cell Biol. 93 (2), 109–118. 10.1139/bcb-2014-0095 25454218

[B15] Cushing L. Kuang P. P. Qian J. Shao F. Wu J. Little F. (2011). miR-29 is a major regulator of genes associated with pulmonary fibrosis. Am. J. Respir. Cell Mol. Biol. 45 (2), 287–294. 10.1165/rcmb.2010-0323OC 20971881 PMC3175558

[B16] Deng Z. He Y. Yang X. Shi H. Shi A. Lu L. (2017). MicroRNA-29: A crucial player in fibrotic disease. Mol. Diagn. Ther. 21 (3), 285–294. 10.1007/s40291-016-0253-9 28130757

[B17] Dinh P.-U. C. Paudel D. Brochu H. Popowski K. D. Gracieux M. C. Cores J. (2020). Inhalation of lung spheroid cell secretome and exosomes promotes lung repair in pulmonary fibrosis. Nat. Commun. 11 (1), 1064. 10.1038/s41467-020-14344-7 32111836 PMC7048814

[B147] Dong H. Dong S. Zhang L. (2016). MicroRNA-214 exerts a cardio-protective effect by inhibition of fibrosis. Anat. Rec. (Hoboken) 299 (10), 1348–1357. 10.1002/ar.23396 27357906

[B18] Fang S. Xu C. Zhang Y. Xue C. Yang C. Bi H. (2016). Umbilical cord-derived mesenchymal stem cell-derived exosomal microRNAs suppress myofibroblast differentiation by inhibiting the transforming growth factor-β/SMAD2 pathway during wound healing. Stem Cells Transl. Med. 5 (10), 1425–1439. 10.5966/sctm.2015-0367 27388239 PMC5031180

[B19] Frith J. E. Kusuma G. D. Carthew J. Li F. Cloonan N. Gomez G. A. (2018). Mechanically-sensitive miRNAs bias human mesenchymal stem cell fate via mTOR signalling. Nat. Commun. 9 (1), 257. 10.1038/s41467-017-02486-0 29343687 PMC5772625

[B20] Fu M. Yin W. Zhang W. Zhu Y. Ni H. Gong L. (2022). MicroRNA-15a inhibits hepatic stellate cell activation and proliferation via targeting SRY-box transcription factor 9. Bioengineered 13 (5), 13011–13020. 10.1080/21655979.2022.2068895 35611752 PMC9276033

[B21] Fujita Y. Araya J. Ito S. Kobayashi K. Kosaka N. Yoshioka Y. (2015). Suppression of autophagy by extracellular vesicles promotes myofibroblast differentiation in COPD pathogenesis. J. Extracell. Vesicles 4, 28388. 10.3402/jev.v4.28388 26563733 PMC4643181

[B22] Fujita Y. Kadota T. Araya J. Ochiya T. Kuwano K. (2018). Clinical application of mesenchymal stem cell-derived extracellular vesicle-based therapeutics for inflammatory lung diseases. J. Clin. Med. 7 (10), 355. 10.3390/jcm7100355 PMC6210470 30322213

[B23] Gan H. Xu X. Bai Y. (2022). Trametes robiniophila represses angiogenesis and tumor growth of lung cancer via strengthening let-7d-5p and targeting NAP1L1. Bioengineered 13 (3), 6698–6710. 10.1080/21655979.2021.2012619 34898380 PMC8973683

[B24] Gao Y. Sun J. Dong C. Zhao M. Hu Y. Jin F. (2020). Extracellular vesicles derived from adipose mesenchymal stem cells alleviate PM2.5-induced lung injury and pulmonary fibrosis. Med. Sci. Monit. 26, e922782. 10.12659/msm.922782 32304204 PMC7191958

[B25] Gasparini P. Cascione L. Landi L. Carasi S. Lovat F. Tibaldi C. (2015). microRNA classifiers are powerful diagnostic/prognostic tools in ALK-EGFR-and KRAS-driven lung cancers. Proc. Natl. Acad. Sci. U. S. A. 112 (48), 14924–14929. 10.1073/pnas.1520329112 26627242 PMC4672770

[B26] Glass D. S. Grossfeld D. Renna H. A. Agarwala P. Spiegler P. DeLeon J. (2022). Idiopathic pulmonary fibrosis: Current and future treatment. Clin. Respir. J. 16 (2), 84–96. 10.1111/crj.13466 35001525 PMC9060042

[B27] Gong X. Wang X. Zhou F. (2020). Liver microRNA-29b-3p positively correlates with relative enhancement values of magnetic resonance imaging and represses liver fibrosis. J. Biochem. 168 (6), 603–609. 10.1093/jb/mvaa074 32653922

[B28] Guiot J. Cambier M. Boeckx A. Henket M. Nivelles O. Gester F. (2020). Macrophage-derived exosomes attenuate fibrosis in airway epithelial cells through delivery of antifibrotic miR-142-3p. Thorax 75 (10), 870–881. 10.1136/thoraxjnl-2019-214077 32759383 PMC7509395

[B29] Guiot J. Struman I. Louis E. Louis R. Malaise M. Njock M.-S. (2019). Exosomal miRNAs in lung diseases: From biologic function to therapeutic targets. J. Clin. Med. 8 (9), 1345. 10.3390/jcm8091345 PMC6781233 31470655

[B30] Guo F. Lin S. C. Zhao M. S. Yu B. Li X. Y. Gao Q. (2017). microRNA-142-3p inhibits apoptosis and inflammation induced by bleomycin through down-regulation of Cox-2 in MLE-12 cells. Braz. J. Med. Biol. Res. 50 (7), e5974. 10.1590/1414-431x20175974 28678919 PMC5496156

[B31] He Y. Huang C. Lin X. Li J. (2013). MicroRNA-29 family, a crucial therapeutic target for fibrosis diseases. Biochimie 95 (7), 1355–1359. 10.1016/j.biochi.2013.03.010 23542596

[B32] Hong Y. Cao H. Wang Q. Ye J. Sui L. Feng J. (2016). MiR-22 may suppress fibrogenesis by targeting TGFβR I in cardiac fibroblasts. Cell. Physiol. biochem. 40 (6), 1345–1353. 10.1159/000453187 27997889

[B33] Hua Y. Ding Y. Hou Y. Liu Y. Mao K. Cui Y. (2021). Exosomal MicroRNA: Diagnostic marker and therapeutic tool for lung diseases. Curr. Pharm. Des. 27 (26), 2934–2942. 10.2174/1381612827666210608150640 34102963

[B34] Huang W. Huang F. Zhang R. Luo H. (2021). LncRNA Neat1 expedites the progression of liver fibrosis in mice through targeting miR-148a-3p and miR-22-3p to upregulate Cyth3. Cell Cycle 20 (5-6), 490–507. 10.1080/15384101.2021.1875665 33550894 PMC8018424

[B35] Huleihel L. Ben-Yehudah A. Milosevic J. Yu G. Pandit K. Sakamoto K. (2014). Let-7d microRNA affects mesenchymal phenotypic properties of lung fibroblasts. Am. J. Physiol. Lung Cell. Mol. Physiol. 306 (6), L534–L542. 10.1152/ajplung.00149.2013 24441869 PMC3949080

[B36] Ideozu J. E. Zhang X. Rangaraj V. McColley S. Levy H. (2019). Microarray profiling identifies extracellular circulating miRNAs dysregulated in cystic fibrosis. Sci. Rep. 9 (1), 15483. 10.1038/s41598-019-51890-7 31664087 PMC6820733

[B37] Inomata M. Kamio K. Azuma A. Matsuda K. Usuki J. Morinaga A. (2021). Rictor-targeting exosomal microRNA-16 ameliorates lung fibrosis by inhibiting the mTORC2-SPARC axis. Exp. Cell Res. 398 (2), 112416. 10.1016/j.yexcr.2020.112416 33307020

[B38] Isobe T. Hisamori S. Hogan D. J. Zabala M. Hendrickson D. G. Dalerba P. (2014). miR-142 regulates the tumorigenicity of human breast cancer stem cells through the canonical WNT signaling pathway. Elife 3, e01977. 10.7554/eLife.01977 PMC4235011 25406066

[B39] Ji D. Li B. Shao Q. Li F. Li Z. Chen G. (2015). MiR-22 suppresses BMP7 in the development of cirrhosis. Cell. Physiol. biochem. 36 (3), 1026–1036. 10.1159/000430276 26112332

[B40] Jin Z. Q. (2021). MicroRNA targets and biomarker validation for diabetes-associated cardiac fibrosis. Pharmacol. Res. 174, 105941. 10.1016/j.phrs.2021.105941 34656765

[B41] Kadota T. Fujita Y. Araya J. Watanabe N. Fujimoto S. Kawamoto H. (2021). Human bronchial epithelial cell-derived extracellular vesicle therapy for pulmonary fibrosis via inhibition of TGF-β-WNT crosstalk. J. Extracell. Vesicles 10 (10), e12124. 10.1002/jev2.12124 34377373 PMC8329991

[B42] Kadota T. Fujita Y. Yoshioka Y. Araya J. Kuwano K. Ochiya T. (2016). Extracellular vesicles in chronic obstructive pulmonary disease. Int. J. Mol. Sci. 17 (11), 1801. 10.3390/ijms17111801 PMC5133802 27801806

[B43] Kadota T. Yoshioka Y. Fujita Y. Araya J. Minagawa S. Hara H. (2020). Extracellular vesicles from fibroblasts induce epithelial cell senescence in pulmonary fibrosis. Am. J. Respir. Cell Mol. Biol. 63 (5), 623–636. 10.1165/rcmb.2020-0002OC 32730709

[B44] Kang J. H. Jung M. Y. Choudhury M. Leof E. B. (2019). Transforming growth factor beta induces fibroblasts to express and release the immunomodulatory protein PD‐L1 into extracellular vesicles. FASEB J. 34 (2), 2213–2226. 10.1096/fj.201902354R 31907984

[B45] Kaur G. Maremanda K. P. Campos M. Chand H. S. Li F. Hirani N. (2021). Distinct Exosomal miRNA profiles from BALF and lung tissue of COPD and IPF patients. Int. J. Mol. Sci. 22 (21), 11830. 10.3390/ijms222111830 34769265 PMC8584050

[B46] Kim J. You G. E. Woo M. Chang N. H. Lee J. (2021). Discovery of lactoferrin as a stimulant for hADSC-derived EV secretion and proof of enhancement of resulting EVs through skin model. Int. J. Mol. Sci. 22 (20), 10993. 10.3390/ijms222010993 34681650 PMC8541114

[B47] Koh Y. Q. Tan C. J. Toh Y. L. Sze S. K. Ho H. K. Limoli C. L. (2020). Role of exosomes in cancer-related cognitive impairment. Int. J. Mol. Sci. 21 (8), E2755. 10.3390/ijms21082755 32326653 PMC7215650

[B48] Kok V. C. Yu C. C. (2020). Cancer-derived exosomes: Their role in cancer biology and biomarker development. Int. J. Nanomedicine 15, 8019–8036. 10.2147/IJN.S272378 33116515 PMC7585279

[B49] Kumar P. Dezso Z. MacKenzie C. Oestreicher J. Agoulnik S. Byrne M. (2013). Circulating miRNA biomarkers for Alzheimer's disease. PLoS One 8 (7), e69807. 10.1371/journal.pone.0069807 23922807 PMC3726785

[B50] Kumar S. Sharawat S. K. Ali A. Gaur V. Malik P. S. Kumar S. (2020). Identification of differentially expressed circulating serum microRNA for the diagnosis and prognosis of Indian non-small cell lung cancer patients. Curr. Probl. Cancer 44 (4), 100540. 10.1016/j.currproblcancer.2020.100540 32007320

[B51] Kuse N. Kamio K. Azuma A. Matsuda K. Inomata M. Usuki J. (2020). Exosome-derived microRNA-22 ameliorates pulmonary fibrosis by regulating fibroblast-to-myofibroblast differentiation *in vitro* and *in vivo* . J. Nippon Med. Sch. 87 (3), 118–128. 10.1272/jnms.JNMS.2020_87-302 31776321

[B52] Lacedonia D. Scioscia G. Soccio P. Conese M. Catucci L. Palladino G. P. (2021). Downregulation of exosomal let-7d and miR-16 in idiopathic pulmonary fibrosis. BMC Pulm. Med. 21 (1), 188. 10.1186/s12890-021-01550-2 34088304 PMC8176704

[B53] Lawrence J. Nho R. (2018). The role of the mammalian target of rapamycin (mTOR) in pulmonary fibrosis. Int. J. Mol. Sci. 19 (3), 778. 10.3390/ijms19030778 PMC5877639 29518028

[B54] Lei X. He N. Zhu L. Zhou M. Zhang K. Wang C. (2020). Mesenchymal stem cell-derived extracellular vesicles attenuate radiation-induced lung injury via miRNA-214-3p. Antioxid. Redox Signal. 35, 849–862. 10.1089/ars.2019.7965 32664737

[B55] Li J. W. Wei L. Han Z. Chen Z. (2019). Mesenchymal stromal cells-derived exosomes alleviate ischemia/reperfusion injury in mouse lung by transporting anti-apoptotic miR-21-5p. Eur. J. Pharmacol. 852, 68–76. 10.1016/j.ejphar.2019.01.022 30682335

[B56] Li Y. Shen Z. Jiang X. Wang Y. Yang Z. Mao Y. (2022). Mouse mesenchymal stem cell-derived exosomal miR-466f-3p reverses EMT process through inhibiting AKT/GSK3β pathway via c-MET in radiation-induced lung injury. J. Exp. Clin. Cancer Res. 41 (1), 128. 10.1186/s13046-022-02351-z 35392967 PMC8988379

[B57] Liang J. N. Zou X. Fang X. H. Xu J. D. Xiao Z. Zhu J. N. (2019). The Smad3-miR-29b/miR-29c axis mediates the protective effect of macrophage migration inhibitory factor against cardiac fibrosis. Biochim. Biophys. Acta. Mol. Basis Dis. 1865 (9), 2441–2450. 10.1016/j.bbadis.2019.06.004 31175931

[B58] Liguori M. Nuzziello N. Introna A. Consiglio A. Licciulli F. D'Errico E. (2018). Dysregulation of microRNAs and target genes networks in peripheral blood of patients with sporadic amyotrophic lateral sclerosis. Front. Mol. Neurosci. 11, 288. 10.3389/fnmol.2018.00288 30210287 PMC6121079

[B59] Lindenbergh M. F. S. Stoorvogel W. (2018). Antigen presentation by extracellular vesicles from professional antigen-presenting cells. Annu. Rev. Immunol. 36 (1), 435–459. 10.1146/annurev-immunol-041015-055700 29400984

[B60] Liu B. Jiang T. Hu X. Liu Z. Zhao L. Liu H. (2018). Downregulation of microRNA-30a in bronchoalveolar lavage fluid from idiopathic pulmonary fibrosis patients. Mol. Med. Rep. 18 (6), 5799–5806. 10.3892/mmr.2018.9565 30365083

[B61] Liu G. Qian M. Chen M. Chen T. Qin S. (2020). miR-21-5p suppresses mitophagy to alleviate hyperoxia-induced acute lung injury by directly targeting PGAM5. Biomed. Res. Int. 2020, 4807254. 10.1155/2020/4807254 33681349 PMC7907750

[B62] Long J. Badal S. S. Wang Y. Chang B. H. Rodriguez A. Danesh F. R. (2013). MicroRNA-22 is a master regulator of bone morphogenetic protein-7/6 homeostasis in the kidney. J. Biol. Chem. 288 (51), 36202–36214. 10.1074/jbc.M113.498634 24163368 PMC3868735

[B64] Lu Y. F. Liu Y. Fu W. M. Xu J. Wang B. Sun Y. X. (2017). Long noncoding RNA H19 accelerates tenogenic differentiation and promotes tendon healing through targeting miR-29b-3p and activating TGF-β1 signaling. FASEB J. 31 (3), 954–964. 10.1096/fj.201600722R 27895107

[B65] Lucchetti D. Santini G. Perelli L. Ricciardi-Tenore C. Colella F. Mores N. (2021). Detection and characterisation of extracellular vesicles in exhaled breath condensate and sputum of COPD and severe asthma patients. Eur. Respir. J. 58 (2), 2003024. 10.1183/13993003.03024-2020 33795323

[B66] Ma Y. Liu X. Long Y. Chen Y. (2022). Emerging therapeutic potential of mesenchymal stem cell-derived extracellular vesicles in chronic respiratory diseases: An overview of recent progress. Front. Bioeng. Biotechnol. 10, 845042. 10.3389/fbioe.2022.845042 35284423 PMC8913891

[B142] Makiguchi T. Yamada M. Yoshioka Y. (2016). Serum extracellular vesicular miR-21-5p is a predictor of the prognosis in idiopathic pulmonary fibrosis. Respir. Res. 17 (1), 110. 10.1186/s12931-016-0427-3 27596748 PMC5011900

[B68] Makler A. Asghar W. (2020). Exosomal biomarkers for cancer diagnosis and patient monitoring. Expert Rev. Mol. diagn. 20 (4), 387–400. 10.1080/14737159.2020.1731308 32067543 PMC7071954

[B69] Mansouri N. Willis G. R. Fernandez-Gonzalez A. Reis M. Nassiri S. Mitsialis S. A. (2019). Mesenchymal stromal cell exosomes prevent and revert experimental pulmonary fibrosis through modulation of monocyte phenotypes. JCI Insight 4 (21), 128060. 10.1172/jci.insight.128060 31581150 PMC6948760

[B70] Markopoulos G. S. Roupakia E. Tokamani M. Vartholomatos G. Tzavaras T. Hatziapostolou M. (2017). Senescence-associated microRNAs target cell cycle regulatory genes in normal human lung fibroblasts. Exp. Gerontol. 96, 110–122. 10.1016/j.exger.2017.06.017 28658612

[B71] Martin-Medina A. Lehmann M. Burgy O. Hermann S. Baarsma H. A. Wagner D. E. (2018). Increased extracellular vesicles mediate WNT5A signaling in idiopathic pulmonary fibrosis. Am. J. Respir. Crit. Care Med. 198 (12), 1527–1538. 10.1164/rccm.201708-1580OC 30044642

[B72] Mendes-Silva A. P. Pereira K. S. Tolentino-Araujo G. T. Nicolau Ede S. Silva-Ferreira C. M. Teixeira A. L. (2016). Shared biologic pathways between alzheimer disease and major depression: A systematic review of microRNA expression studies. Am. J. Geriatr. Psychiatry 24 (10), 903–912. 10.1016/j.jagp.2016.07.017 27591915

[B73] Merckx G. Hosseinkhani B. Kuypers S. Deville S. Irobi J. Nelissen I. (2020). Angiogenic effects of human dental pulp and bone marrow-derived mesenchymal stromal cells and their extracellular vesicles. Cells 9 (2), 312. 10.3390/cells9020312 PMC7072370 32012900

[B74] Min H. Fan S. Song S. Zhuang Y. Li H. Wu Y. (2016). Plasma microRNAs are associated with acute exacerbation in idiopathic pulmonary fibrosis. Diagn. Pathol. 11 (1), 135. 10.1186/s13000-016-0583-2 27881157 PMC5120520

[B75] Min H. Ma D. Zou W. Wu Y. Ding Y. Zhu C. (2020). FLCN-regulated miRNAs suppressed reparative response in cells and pulmonary lesions of Birt-Hogg-Dubé syndrome. Thorax 75 (6), 476–485. 10.1136/thoraxjnl-2019-213225 32184379 PMC7279199

[B140] Mullenbrock S. Liu F. Szak S. (2018). Systems analysis of transcriptomic and proteomic profiles identifies novel regulation of fibrotic programs by miRNAs in pulmonary fibrosis fibroblasts. Genes (Basel) 9 (12), 588. 10.3390/genes9120588 PMC6316743 30501089

[B76] Neri T. Lombardi S. Faìta F. Petrini S. Balìa C. Scalise V. (2016). Pirfenidone inhibits p38-mediated generation of procoagulant microparticles by human alveolar epithelial cells. Pulm. Pharmacol. Ther. 39, 1–6. 10.1016/j.pupt.2016.05.003 27237042

[B77] Njock M.-S. Guiot J. Henket M. A. Nivelles O. Thiry M. Dequiedt F. (2019). Sputum exosomes: Promising biomarkers for idiopathic pulmonary fibrosis. Thorax 74 (3), 309–312. 10.1136/thoraxjnl-2018-211897 30244194 PMC6467246

[B143] Novelli F. Neri T. Tavanti L. (2014). Procoagulant, tissue factor-bearing microparticles in bronchoalveolar lavage of interstitial lung disease patients: An observational study. PLoS One 9 (4), e95013. 10.1371/journal.pone.0095013 24777000 PMC4002423

[B78] O'Neill H. C. Quah B. J. (2008). Exosomes secreted by bacterially infected macrophages are proinflammatory. Sci. Signal. 1 (6), pe8. 10.1126/stke.16pe8 18272468

[B79] Ostrowski M. Carmo N. B. Krumeich S. Fanget I. Raposo G. Savina A. (2010). Rab27a and Rab27b control different steps of the exosome secretion pathway. Nat. Cell Biol. 12 (1), 19–30. sup pp 1-13. 10.1038/ncb2000 19966785

[B80] Pan B. T. Teng K. Wu C. Adam M. Johnstone R. M. (1985). Electron microscopic evidence for externalization of the transferrin receptor in vesicular form in sheep reticulocytes. J. Cell Biol. 101 (3), 942–948. 10.1083/jcb.101.3.942 2993317 PMC2113705

[B81] Pandit K. V. Milosevic J. Kaminski N. (2011). MicroRNAs in idiopathic pulmonary fibrosis. Transl. Res. 157 (4), 191–199. 10.1016/j.trsl.2011.01.012 21420029

[B82] Parimon T. Yao C. Habiel D. M. Ge L. Bora S. A. Brauer R. (2019). Syndecan-1 promotes lung fibrosis by regulating epithelial reprogramming through extracellular vesicles. JCI Insight 4 (17), 129359. 10.1172/jci.insight.129359 PMC6777916 31393853

[B83] Peng L. Chen Y. Shi S. Wen H. (2022). Stem cell-derived and circulating exosomal microRNAs as new potential tools for diabetic nephropathy management. Stem Cell Res. Ther. 13 (1), 25. 10.1186/s13287-021-02696-w 35073973 PMC8785577

[B84] Peng X. Yang L. Liu H. Pang S. Chen Y. Fu J. (2016). Identification of circulating microRNAs in biliary atresia by next-generation sequencing. J. Pediatr. Gastroenterol. Nutr. 63 (5), 518–523. 10.1097/mpg.0000000000001194 26960174

[B85] Phinney D. G. Di Giuseppe M. Njah J. Sala E. Shiva S. St Croix C. M. (2015). Mesenchymal stem cells use extracellular vesicles to outsource mitophagy and shuttle microRNAs. Nat. Commun. 6, 8472. 10.1038/ncomms9472 26442449 PMC4598952

[B86] Purghè B. Manfredi M. Ragnoli B. Baldanzi G. Malerba M. (2021). Exosomes in chronic respiratory diseases. Biomed. Pharmacother. 144, 112270. 10.1016/j.biopha.2021.112270 34678722

[B87] Qi A. Wang T. Li W. Wang Y. Chai Y. (2021). The effect of miR-21-5p on the MAP2K3 expressions and cellular apoptosis in the lung tissues of neonatal rats with hyperoxia-induced lung injuries. Am. J. Transl. Res. 13 (4), 2784–2793. 34017441 PMC8129236

[B88] Qin S. Wang H. Liu G. Mei H. Chen M. (2019). miR-21-5p ameliorates hyperoxic acute lung injury and decreases apoptosis of AEC II cells via PTEN/AKT signaling in rats. Mol. Med. Rep. 20 (6), 4953–4962. 10.3892/mmr.2019.10779 31702805 PMC6854583

[B90] Ramachandra L. Qu Y. Wang Y. Lewis C. J. Cobb B. A. Takatsu K. (2010). *Mycobacterium tuberculosis* synergizes with ATP to induce release of microvesicles and exosomes containing major histocompatibility complex class II molecules capable of antigen presentation. Infect. Immun. 78 (12), 5116–5125. 10.1128/iai.01089-09 20837713 PMC2981298

[B144] Rawal S. Munasinghe P. E. Nagesh P. T. (2017). Down-regulation of miR-15a/b accelerates fibrotic remodelling in the Type 2 diabetic human and mouse heart. Clin. Sci. (Lond) 131 (9), 847–863. 10.1042/CS2016091 28289072

[B91] Ren W. Hou J. Yang C. Wang H. Wu S. Wu Y. (2019). Extracellular vesicles secreted by hypoxia pre-challenged mesenchymal stem cells promote non-small cell lung cancer cell growth and mobility as well as macrophage M2 polarization via miR-21-5p delivery. J. Exp. Clin. Cancer Res. 38 (1), 62. 10.1186/s13046-019-1027-0 30736829 PMC6367822

[B92] Richeldi L. Collard H. R. Jones M. (2017). Idiopathic pulmonary fibrosis. Lancet 389 (10082), 1941–1952. 10.1016/S0140-6736(17)30866-8 28365056

[B93] Rogler C. E. Matarlo J. S. Kosmyna B. Fulop D. Rogler L. E. (2017). Knockdown of miR-23, miR-27, and miR-24 alters fetal liver development and blocks fibrosis in mice. Gene Expr. 17 (2), 99–114. 10.3727/105221616x693891 27938504 PMC8751183

[B94] Shentu T.-P. Huang T.-S. Cernelc-Kohan M. Chan J. Wong S. S. Espinoza C. R. (2017). Thy-1 dependent uptake of mesenchymal stem cell-derived extracellular vesicles blocks myofibroblastic differentiation. Sci. Rep. 7 (1), 18052. 10.1038/s41598-017-18288-9 29273797 PMC5741716

[B95] Shi J. Li F. Luo M. Wei J. Liu X. (2017). Distinct roles of Wnt/β-catenin signaling in the pathogenesis of chronic obstructive pulmonary disease and idiopathic pulmonary fibrosis. Mediat. Inflamm. 2017, 3520581. 10.1155/2017/3520581 PMC5447271 28588349

[B96] Shi L. Ren J. Li J. Wang D. Wang Y. Qin T. (2021). Extracellular vesicles derived from umbilical cord mesenchymal stromal cells alleviate pulmonary fibrosis by means of transforming growth factor-β signaling inhibition. Stem Cell Res. Ther. 12 (1), 230. 10.1186/s13287-021-02296-8 33845892 PMC8041243

[B97] Song Y. Zuo Y. Qian X. L. Chen Z. P. Wang S. K. Song L. (2017). Inhibition of microRNA-21-5p promotes the radiation sensitivity of non-small cell lung cancer through HMSH2. Cell. Physiol. biochem. 43 (3), 1258–1272. 10.1159/000481839 29024929

[B98] Sun J. Su W. Zhao X. Shan T. Jin T. Guo Y. (2019). LncRNA PFAR contributes to fibrogenesis in lung fibroblasts through competitively binding to miR-15a. Biosci. Rep. 39 (7), BSR20190280. 10.1042/bsr20190280 31273058 PMC6639460

[B99] Sun L. Sun M. Ma K. Liu J. (2020). Let-7d-5p suppresses inflammatory response in neonatal rats with necrotizing enterocolitis via LGALS3-mediated TLR4/NF-κB signaling pathway. Am. J. Physiol. Cell Physiol. 319 (6), C967–C979. 10.1152/ajpcell.00571.2019 32667865

[B146] Sun M. Yu H. Zhang Y. Li Z. Gao W. (2015). MicroRNA-214 mediates isoproterenol-induced proliferation and collagen synthesis in cardiac fibroblasts. Sci Rep. 5, 18351. 10.1038/srep1835 26692091 PMC4686919

[B100] Sun L. Zhu M. Feng W. Lin Y. Yin J. Jin J. (2019). Exosomal miRNA Let-7 from menstrual blood-derived endometrial stem cells alleviates pulmonary fibrosis through regulating mitochondrial dna damage. Oxid. Med. Cell. Longev. 2019, 4506303. 10.1155/2019/4506303 31949877 PMC6948326

[B139] Szul T. Bratcher P. E. Fraser K. B. (2016). Toll-like receptor 4 engagement mediates prolyl endopeptidase release from airway epithelia via exosomes. Am. J. Respir. Cell Mol. Biol. 54 (3), 359–369. 10.1165/rcmb.2015-0108OC 26222144 PMC5455678

[B101] Tamai K. Tanaka N. Nakano T. Kakazu E. Kondo Y. Inoue J. (2010). Exosome secretion of dendritic cells is regulated by Hrs, an ESCRT-0 protein. Biochem. Biophys. Res. Commun. 399 (3), 384–390. 10.1016/j.bbrc.2010.07.083 20673754

[B145] Tan J. L. Lau S. N. Leaw B. (2018). Amnion epithelial cell-derived exosomes restrict lung injury and enhance endogenous lung repair. Stem Cells Transl. Med. 7 (2), 180–196. 10.1002/sctm.17-0185 29297621 PMC5788876

[B102] Tang J. Li X. Cheng T. Wu J. (2021). miR-21-5p/SMAD7 axis promotes the progress of lung cancer. Thorac. Cancer 12 (17), 2307–2313. 10.1111/1759-7714.14060 34254453 PMC8410517

[B103] Tao R. Fan X. X. Yu H. J. Ai G. Zhang H. Y. Kong H. Y. (2018). MicroRNA-29b-3p prevents Schistosoma japonicum-induced liver fibrosis by targeting COL1A1 and COL3A1. J. Cell. Biochem. 119 (4), 3199–3209. 10.1002/jcb.26475 29091295

[B104] Tasena H. Faiz A. Timens W. Noordhoek J. Hylkema M. N. Gosens R. (2018). microRNA-mRNA regulatory networks underlying chronic mucus hypersecretion in COPD. Eur. Respir. J. 52 (3), 1701556. 10.1183/13993003.01556-2017 30072506

[B105] Tijsen A. J. van der Made I. van den Hoogenhof M. M. Wijnen W. J. van Deel E. D. de Groot N. E. (2014). The microRNA-15 family inhibits the TGFβ-pathway in the heart. Cardiovasc. Res. 104 (1), 61–71. 10.1093/cvr/cvu184 25103110

[B106] Trappe A. Donnelly S. C. McNally P. Coppinger J. A. (2021). Role of extracellular vesicles in chronic lung disease. Thorax 76 (10), 1047–1056. 10.1136/thoraxjnl-2020-216370 33712504 PMC8461402

[B107] Wan X. Chen S. Fang Y. Zuo W. Cui J. Xie S. (2020). Mesenchymal stem cell‐derived extracellular vesicles suppress the fibroblast proliferation by downregulating FZD6 expression in fibroblasts via micrRNA‐29b‐3p in idiopathic pulmonary fibrosis. J. Cell. Physiol. 235 (11), 8613–8625. 10.1002/jcp.29706 32557673

[B108] Wang A. P. Li X. H. Gong S. X. Li W. Q. Hu C. P. Zhang Z. (2015). miR-100 suppresses mTOR signaling in hypoxia-induced pulmonary hypertension in rats. Eur. J. Pharmacol. 765, 565–573. 10.1016/j.ejphar.2015.09.031 26409044

[B109] Wang H. Liu Y. Wang D. Xu Y. Dong R. Yang Y. (2019). The upstream pathway of mTOR-mediated autophagy in liver diseases. Cells 8 (12), E1597. 10.3390/cells8121597 31835352 PMC6953127

[B110] Wang J. Hu L. Huang H. Yu Y. Wang J. Yu Y. (2020). CAR (CARSKNKDC) peptide modified ReNcell-derived extracellular vesicles as a novel therapeutic agent for targeted pulmonary hypertension therapy. Hypertension 76 (4), 1147–1160. 10.1161/HYPERTENSIONAHA.120.15554 32829668

[B111] Wang J. He F. Chen L. Li Q. Jin S. Zheng H. (2018). Resveratrol inhibits pulmonary fibrosis by regulating miR-21 through MAPK/AP-1 pathways. Biomed. Pharmacother. 105, 37–44. 10.1016/j.biopha.2018.05.104 29843043

[B112] Wang Y. C. Xie H. Zhang Y. C. Meng Q. H. Xiong M. M. Jia M. W. (2021). Exosomal miR-107 antagonizes profibrotic phenotypes of pericytes by targeting a pathway involving HIF-1α/Notch1/PDGFRβ/YAP1/Twist1 axis *in vitro* . Am. J. Physiol. Heart Circ. Physiol. 320 (2), H520–H534. 10.1152/ajpheart.00373.2020 33216617

[B113] Wang Y. Ouyang M. Wang Q. Jian Z. (2016). MicroRNA-142-3p inhibits hypoxia/reoxygenation-induced apoptosis and fibrosis of cardiomyocytes by targeting high mobility group box 1. Int. J. Mol. Med. 38 (5), 1377–1386. 10.3892/ijmm.2016.2756 28025989 PMC5065300

[B114] Wang Y. Zhong Y. Sun K. Fan Y. Liao J. Wang G. (2021). Identification of exosome miRNAs in bronchial epithelial cells after PM2.5 chronic exposure. Ecotoxicol. Environ. Saf. 215, 112127. 10.1016/j.ecoenv.2021.112127 33714894

[B115] Wu J. Kuang L. Chen C. Yang J. Zeng W. N. Li T. (2019). miR-100-5p-abundant exosomes derived from infrapatellar fat pad MSCs protect articular cartilage and ameliorate gait abnormalities via inhibition of mTOR in osteoarthritis. Biomaterials 206, 87–100. 10.1016/j.biomaterials.2019.03.022 30927715

[B116] Wu Y. Zhang Z. Li J. Zhong H. Yuan R. Deng Z. (2022). Mechanism of adipose-derived mesenchymal stem cell-derived extracellular vesicles carrying miR-21-5p in hyperoxia-induced lung injury. Stem Cell Rev. Rep. 18 (3), 1007–1024. 10.1007/s12015-021-10311-x 34882302

[B117] Xie H. Gao Y. M. Zhang Y. C. Jia M. W. Peng F. Meng Q. H. (2020). Low let-7d exosomes from pulmonary vascular endothelial cells drive lung pericyte fibrosis through the TGFβRI/FoxM1/Smad/β-catenin pathway. J. Cell. Mol. Med. 24 (23), 13913–13926. 10.1111/jcmm.15989 33179861 PMC7753874

[B118] Xie L. Zeng Y. (2020). Therapeutic potential of exosomes in pulmonary fibrosis. Front. Pharmacol. 11, 590972. 10.3389/fphar.2020.590972 33343360 PMC7746877

[B119] Xie T. Liang J. Guo R. Liu N. Noble P. W. Jiang D. (2011). Comprehensive microRNA analysis in bleomycin-induced pulmonary fibrosis identifies multiple sites of molecular regulation. Physiol. Genomics 43 (9), 479–487. 10.1152/physiolgenomics.00222.2010 21266501 PMC3110895

[B120] Xue Y. Fan X. Yang R. Jiao Y. Li Y. (2020). miR-29b-3p inhibits post-infarct cardiac fibrosis by targeting FOS. Biosci. Rep. 40 (9), BSR20201227. 10.1042/bsr20201227 32812641 PMC7468097

[B121] Xunian Z. Kalluri R. (2020). Biology and therapeutic potential of mesenchymal stem cell-derived exosomes. Cancer Sci. 111 (9), 3100–3110. 10.1111/cas.14563 32639675 PMC7469857

[B122] Yamada M. (2021). Extracellular vesicles: Their emerging roles in the pathogenesis of respiratory diseases. Respir. Investig. 59 (3), 302–311. 10.1016/j.resinv.2021.02.006 33753011

[B123] Yamada M. (2020). The roles of microRNAs and extracellular vesicles in the pathogeneses of idiopathic pulmonary fibrosis and acute respiratory distress syndrome. Tohoku J. Exp. Med. 251 (4), 313–326. 10.1620/tjem.251.313 32779621

[B124] Yan L. Ma J. Wang Y. Zan J. Wang Z. Zhu Y. (2018). miR-21-5p induces cell proliferation by targeting TGFBI in non-small cell lung cancer cells. Exp. Ther. Med. 16 (6), 4655–4663. 10.3892/etm.2018.6752 30542417 PMC6257667

[B125] Yang L. Fan Y. Zhang X. Ma J. (2017). miRNA-23 regulates high glucose induced epithelial to mesenchymal transition in human mesotheial peritoneal cells by targeting VDR. Exp. Cell Res. 360 (2), 375–383. 10.1016/j.yexcr.2017.09.029 28942023

[B126] Yang X. Dan X. Men R. Ma L. Wen M. Peng Y. (2017). MiR-142-3p blocks TGF-β-induced activation of hepatic stellate cells through targeting TGFβRI. Life Sci. 187, 22–30. 10.1016/j.lfs.2017.08.017 28823564

[B127] Yang Z. Xu X. Song C. (2022). Circular RNA La-Related Protein 4 inhibits non-small cell lung cancer cell proliferation while promotes apoptosis through sponging microRNA-21-5p. Cancer biother. Radiopharm. 37 (2), 111–118. 10.1089/cbr.2020.3707 32614609

[B128] Yao M.-Y. Zhang W.-H. Ma W.-T. Liu Q.-H. Xing L.-H. Zhao G.-F. (2019). MicroRNA-328 in exosomes derived from M2 macrophages exerts a promotive effect on the progression of pulmonary fibrosis via FAM13A in a rat model. Exp. Mol. Med. 51 (6), 1–16. 10.1038/s12276-019-0255-x PMC6547742 31164635

[B129] Ye X. Luo H. Chen Y. Wu Q. Xiong Y. Zhu J. (2015). MicroRNAs 99b-5p/100-5p regulated by endoplasmic reticulum stress are involved in Abeta-induced pathologies. Front. Aging Neurosci. 7, 210. 10.3389/fnagi.2015.00210 26635599 PMC4649061

[B130] Zhang Y. Zhao S. Wu D. Liu X. Shi M. Wang Y. (2018). MicroRNA-22 promotes renal tubulointerstitial fibrosis by targeting PTEN and suppressing autophagy in diabetic nephropathy. J. Diabetes Res. 2018, 4728645. 10.1155/2018/4728645 29850604 PMC5903315

[B131] Zhao X. S. Ren Y. Wu Y. Ren H. K. Chen H. (2020). MiR-30b-5p and miR-22-3p restrain the fibrogenesis of post-myocardial infarction in mice via targeting PTAFR. Eur. Rev. Med. Pharmacol. Sci. 24 (7), 3993–4004. 10.26355/eurrev_202004_20869 32329883

[B132] Zheng H. Li W. Wang Y. Xie T. Cai Y. Wang Z. (2014). miR-23a inhibits E-cadherin expression and is regulated by AP-1 and NFAT4 complex during Fas-induced EMT in gastrointestinal cancer. Carcinogenesis 35 (1), 173–183. 10.1093/carcin/bgt274 23929433

[B133] Zheng J. Yu L. Chen W. Lu X. Fan X. (2018). Circulating exosomal microRNAs reveal the mechanism of Fructus Meliae Toosendan-induced liver injury in mice. Sci. Rep. 8 (1), 2832. 10.1038/s41598-018-21113-6 29434260 PMC5809479

[B134] Zhou X. Liu H. Pang Y. Wang M. Liu S. (2022). UTMD-mediated delivery of miR-21-5p inhibitor suppresses the development of lung cancer. Tissue Cell 74, 101719. 10.1016/j.tice.2021.101719 34979378

[B135] Zhou Y. Gao Y. Zhang W. Chen Y. Jin M. Yang Z. (2021). Exosomes derived from induced pluripotent stem cells suppresses M2-type macrophages during pulmonary fibrosis via miR-302a-3p/TET1 axis. Int. Immunopharmacol. 99, 108075. 10.1016/j.intimp.2021.108075 34435585

[B136] Zhou Y. Lv X. Qu H. Zhao K. Fu L. Zhu L. (2018). Preliminary screening and functional analysis of circular RNAs associated with hepatic stellate cell activation. Gene 677, 317–323. 10.1016/j.gene.2018.08.052 30118889

[B137] Zhu G. H. Li R. Zeng Y. Zhou T. Xiong F. Zhu M. (2018). MicroRNA-142-3p inhibits high-glucose-induced endothelial-to-mesenchymal transition through targeting TGF-β1/Smad pathway in primary human aortic endothelial cells. Int. J. Clin. Exp. Pathol. 11 (3), 1208–1217. 31938215 PMC6958121

[B138] Zhu J. Zhang J. Ji X. Tan Z. Lubman D. M. (2021). Column-based technology for CD9-HPLC immunoaffinity isolation of serum extracellular vesicles. J. Proteome Res. 20 (10), 4901–4911. 10.1021/acs.jproteome.1c00549 34473505 PMC8496948

